# Atorvastatin Alleviates Experimental Diabetic Cardiomyopathy by Regulating the GSK-3β-PP2Ac-NF-κB Signaling Axis

**DOI:** 10.1371/journal.pone.0166740

**Published:** 2016-11-16

**Authors:** Xiao-min Ren, Guang-feng Zuo, Wen Wu, Jie Luo, Peng Ye, Shao-liang Chen, Zuo-ying Hu

**Affiliations:** Department of Cardiology, Nanjing First Hospital, Nanjing Medical University, Qinhuai, Nanjing 210006, P.R. China; University of PECS Medical School, HUNGARY

## Abstract

Recent studies reported that atorvastatin (ATOR) alleviated progression of experimental diabetic cardiomyopathy (DCM), possibly by protecting against apoptosis. However, the underlying mechanisms of this protective effect remain unclear. Therefore, our study investigated the role of the glycogen synthase kinase (GSK)-3β-protein phosphatase 2A(PP2A)-NF-κB signaling pathway in the anti-apoptotic and cardioprotective effects of ATOR on cardiomyocytes cultured in high glucose (HG) and in DCM. Our results showed that, in HG-cultured cardiomyocytes, phosphorylation of GSK-3β was decreased, while that of the PP2A catalytic subunit C (PP2Ac) and IKK/IкBα was increased, followed by NF-кB nuclear translocation and apoptosis. IKK/IкBα phosphorylation and NF-кB nuclear translocation were also increased by treatment of cells with okadaic acid (OA), a selective PP2A inhibitor, or by silencing PP2Ac expression. The opposite results were obtained by silencing GSK-3β expression, which resulted in PP2Ac activation. Furthermore, IKK/IкBα phosphorylation and NF-кB nuclear translocation were markedly inhibited and apoptosis attenuated in cells treated with ATOR. These effects occurred through inactivation of GSK-3β and subsequent activation of PP2Ac. They were abolished by treatment of cells with OA or PP2Ac siRNA. In mice with type 1 diabetes mellitus, treatment with ATOR, at 10 mg-kg^−1^-d^−1^, significantly suppressed GSK-3β activation, IKK/IкBα phosphorylation, NF-кB nuclear translocation and caspase-3 activation, while also activating PP2Ac. Finally, improvements in histological abnormalities, fibrosis, apoptosis and cardiac dysfunction were observed in diabetic mice treated with ATOR. These findings demonstrated that ATOR protected against HG-induced apoptosis in cardiomyocytes and alleviated experimental DCM by regulating the GSK-3β-PP2A-NF-κB signaling pathway.

## Introduction

Diabetic cardiomyopathy (DCM) is one of the most prevalent complications in patients with diabetes and often occurs independently of coronary artery disease, hypertension or other cardiovascular diseases [1, 2]. In recent decades, the prevalence of diabetes increased significantly and more than half of diabetes cases were concomitant with DCM [3, 4]. Because DCM contributes to morbidity and mortality [5, 6], hindering its development is necessary for its treatment.

Previous studies showed that cardiomyocyte apoptosis played a crucial role in pathogenesis of DCM [7]. Increased cardiomyocyte apoptosis was a predominant cause of loss of contractile tissue, cardiac remodeling and, eventually, dysfunction [8]. Our previous study showed that inhibition of cardiomyocyte apoptosis improved cardiac function in diabetic mice [9]. Thus, anti-apoptosis is a potential treatment strategy for DCM.

Recent studies showed that sustained activation of the NF-кB signaling pathway initiated apoptosis in HG-cultured cardiomyocytes [8, 10] and inhibition of this pathway improved cardiac dysfunction in diabetic mice [8]. Thus, targeted inhibition of persistent NF-κB signaling activation might effectively treat DCM. However, the underlying mechanisms of this persistent activation are incompletely understood.

Nizamutdinova *et al* [10] found that suppressed PP2Ac activation contributed to sustained IKK/IкBα phosphorylation and subsequent NF-кB nuclear translocation, initiating apoptosis in HG-treated cardiomyocytes. It is interesting to note that PP2Ac inactivation, resulting from activated GSK-3β, was reported in both human embryonic kidney 293 (HEK293) cells and neuro2a (N2a) cells [11, 12]. Increased GSK-3β activity was found in diabetes and in insulin resistance [13]. Therefore, activated GSK-3β might lead to inactivated PP2Ac and the subsequent sustained phosphorylation of IKK/IкBα and NF-кB nuclear translocation. Eventually this can lead to generation of apoptosis and development of DCM. Drugs inhibiting GSK-3β might, therefore, be potentially useful agents for treating DCM.

Atorvastatin (ATOR) is an inhibitor of hydroxymethylglutaryl-coenzyme A (HMG-CoA) reductase and is used as a cholesterol-lowering medication. A recent study reported that ATOR alleviated progression of experimental DCM, possibly because of its protective effects against apoptosis, independent of LDL-cholesterol-lowering [14]. Furthermore, Jin *et al* [15] found that ATOR enhanced neurite outgrowth in cultured cortical neurons by inactivating GSK-3β. Thus, we hypothesized that the anti-apoptotic and cardioprotective effects of ATOR on cardiomyocytes cultured in HG and in an experimental model of DCM might depend upon inactivation of GSK-3β and subsequent activation of PP2Ac, as well as on suppression of the sustained activation of the NF-κB signaling pathway. This study was designed to elucidate these underlying mechanisms of the effects of ATOR on cardiomyocytes.

## Materials and Methods

### Cell culture and treatment

H9C2 cells, a clonal line derived from rat heart, were obtained from the Shanghai Institute of Biochemistry and Cell Biology (Shanghai, People’s Republic of China). Cells were cultured in Dulbecco’s modified Eagle's medium (DMEM) (GIBCO-BRL, Rockville, MD, USA), containing 5.5 mmol-L^−1^ D-glucose and supplemented with 10% fetal bovine serum (FBS, GIBCO-BRL), 100 unit-mL^−1^ penicillin and 0.1 mg-mL^−1^ streptomycin, in a humidified incubator at 37°C equilibrated with 5% CO_2_, 95% air. Cells in the HG-treated group were first incubated in normal glucose (5.5 mmol-L^−1^) with minimal essential medium for 12 h, as previously described [16], and then exposed to DMEM containing 33 mmol-L^−1^ D-glucose for various periods.

### Preparation of isolated neonatal rat cardiomyocytes

Sprague Dawley rat pups (1 to 3 days old) were decapitated before the hearts were removed [17]. Primary cultures of neonatal rat cardiomyocytes were prepared using a method published previously [18]. Culture conditions for the cells were similar to those for H9C2 cells. After 3 days, cells were incubated in serum-free essential medium overnight before being treated with the indicated agents.

### Cell apoptosis measurements

Apoptotic cardiomyocytes were detected using an annexin V-FITC and propidium iodide (PI) double-staining assay, according to the manufacturer’s protocol for the Annexin V-FITC/Propidium Iodide Apoptosis Detection kit (BD Bioscience, San Jose, CA, USA) and fluorescence was detected using a flow cytometer (BD Bioscience), as previously described [19].

### Small interfering RNA (siRNA) transfection of cardiomyocytes

All siRNAs were obtained from Dharmacon (Lafayette, CO, USA). Cardiomyocytes were transfected with 50 nM GSK-3β siRNA or 50 nM PP2Ac siRNA oligoribonucleotides, using Lipofectamine RNAiMAX Reagent (Invitrogen, Carlsbad, CA, USA), according to the manufacturer’s instructions. Scrambled probe was used as a negative control. At 24 h after transfection, the normal glucose-containing medium was replaced with HG medium. At 48 h after transfection, cells were used for further analyses.

### Dual luciferase reporter assay

Effects of HG on transcriptional activity of NF-κB in H9C2 cells were determined by a dual luciferase reporter assay. The pGL4.32[luc2P/NF-кB-RE/Hygro] vector (NF-κB luciferase reporter vector, 0.5 μg) (Promega, Madison, WI, USA) and pRL-TK vector (internal Renilla luciferase control vector, 0.05 μg) (Promega) were co-transfected with Lipofectamine 2000 (Invitrogen) according to the manufacturer's instructions. Dual luciferase activity was determined using one reaction plate sequentially, as previously described [16].

### Immunofluorescence assay

Cardiomyocytes were washed three times with ice-cold PBS and fixed with 4% paraformaldehyde at room temperature for 30 min, as previously described [16]. Then cells were washed 3 times with PBS and permeabilized with 0.5% Triton X-100 for 10 min at room temperature. Subsequently, cells were blocked with PBS containing 5% bovine serum albumin (BSA) at room temperature for 30 min, followed by incubation with primary NF-κB p65 antibody (1:25) overnight at 4°C and then with anti-FITC antibody (1:100, green) at room temperature for 2 h. Cardiomyocyte nuclei were counterstained with DAPI (blue). Finally, immunostained cardiomyocytes were visualized by confocal laser scan microscopy coupled with an image analysis system.

### Extraction of cytoplasmic and nuclear proteins

Cytoplasmic and nuclear proteins were extracted from cardiomyocytes. All procedures were performed, according to the manufacturer’s instructions, with NE-PER Nuclear and Cytoplasmic Extraction Reagents (Pierce Biotechnology, Inc., Rockford, IL, USA). NF-κB levels in the cytosolic and nuclear fractions were determined by western blotting. Anti-GAPDH (cytosol extraction) or anti-histone (nuclear extraction) antibodies were used to verify equal sample loading [10].

### Animal studies

Protocols used for all animal experiments were approved by the Institutional Animal Care and Use Committee of Nanjing Medical University (Nanjing, China). Male C57BL/6 mice (8 wk old, 18–22 g) were obtained from the Animal Center of Nanjing Medical University. All animals were maintained on a 12/12 h light/dark cycle at 23 ± 2°C at room temperature with free access to standard chow and water. Diabetes mellitus was induced by daily intraperitoneal (i.p) injections of 55 mg/kg STZ (Sigma-Aldrich, St. Louis, MO,USA, dissolved in 0.1 mol/L citrate buffer, pH 4.5) for 5 consecutive days [18]. Only mice with blood glucose levels ≥16.7 mmol-L^−1^ at 7 d after STZ injection were considered to be diabetic and used in this study. In addition, body weights and blood glucose levels were recorded on days 7, 17, 27, 47, 57 and 67 after STZ induction using previously published methods [8]. The mice were randomly divided into 4 groups: (1) control mice (n = 15); (2) atorvastatin calcium (Pfizer, New York, NY, USA) treated normal mice (Control+ATOR) (n = 15); (3) diabetic mice (DM) (n = 12); and (4) diabetic mice treated with atorvastatin calcium (DM+ATOR) (n = 10). Mice in Control+ATOR and DM+ATOR groups were treated with atorvastatin calcium via ALZET osmotic mini pumps (model 2004, Durect, Cupertino, CA, USA) at the rate of 10 mg-kg^−1^ daily. The osmotic mini pumps were implanted subcutaneously on the back of each mouse, as described in the manufacturer’s instructions. These mice were treated for 12 wk, beginning at 7 d after STZ injection. In parallel, mice in the Control+ATOR group received analogous treatments to detect potential side effects of atorvastatin calcium or osmotic mini pumps. At the end of the animal experiment, the cardiac functions of all mice were measured by echocardiography and blood plasma collected for biochemical analysis. Then, the hearts were excised for western blotting, histopathological examination and TUNEL assay.

### Serum lipid profile

Triglycerides (TG), total cholesterol (TC), low density lipoprotein-cholesterol (LDL-C) and high density lipoprotein-cholesterol (HDL-C) were determined with an Automatic Chemistry Analyzer (HITACHI 7100, Japan) in the Animal Core Facility of Nanjing Medical University, according to the manufacturer's instructions.

### Cardiac hemodynamic characterization

To assess cardiac function, transthoracic echocardiography was performed in mice, using the Vevo2100 system (Fujifilm VisualSonics, Inc., Toronto, Canada) with a high-frequency (30MHz) MS-400 transducer in the Animal Core Facility of Nanjing Medical University. The mice were anesthetized by isoflurane inhalation. An induction with 3% isoflurane, followed by maintenance at 1.5%, was used prior to echocardiography. The parameters measured included heart rate (HR), interventricular septum thickness in diastole (IVS, d) and systole (IVS, s), left ventricle (LV) internal dimension (LVID) in diastole (LVID, d) and systole (LVID, s), LV posterior wall thickness in diastole (LVPW, d) and systole (LVPW, s), LV volume in diastole (LV vol, d) and systole (LV vol, s), LV mass and LV mass corrected (LV mass, Co), peak E:A ratio (E/A). LV fractional shortening percentage (FS%) = [(LVID, d − LVID, s)/LVID, d]×100; LV ejection fraction percentage (EF%) = [(LV end-diastolic volume − LV end-systolic volume)/LV end-diastolic volume]×100 [8].

### Western blot analysis

Heart samples were homogenized in lysis buffer containing protease and phosphatase inhibitors. Cultured H9C2 cells were lysed in RIPA buffer, as previously described [16]. The concentrations of soluble protein samples were determined with the Pierce BCA protein assay (Thermofisher Scientific, Massachusetts, MA, USA) with BSA as the standard. Equal amounts of total extracted protein (20–80 μg) were separated by 10% or 12% gradient sodium dodecyl sulfate-polyacrylamide gel electrophoresis (SDS-PAGE) and transferred to polyvinylidene difluoride (PVDF) membranes. Nonspecific protein binding was prevented by incubation of membranes in blocking buffer (5% skim milk, 20 mM Tris–HCl, pH 7.6, 150 mM NaCl and 0.05% Tween-20) for 2 h at room temperature. Subsequently, membranes were incubated with specific primary antibodies against GAPDH, caspase-3, cleaved caspase-3, Bax, Bcl-2, phospho-NF-κB, NF-κB, phospho-IKK(Ser176/180), PP2Ac, phospho-GSK-3β(Ser9) (Cell Signaling Technology Inc., Beverly, MA, USA), histone, phospho-IκBα(Ser32/36), IκBα, IKKα (Santa Cruz Biotechnology Inc., Santa Cruz, CA) or phospho-PP2Ac(Y307) (Abcam, Cambridge, UK) in the blocking buffer, with gentle shaking at 4°C overnight. Next, membranes were washed in Tris-buffered saline with Tween (1×TBST) 3 times for 15 min each. Subsequently, membranes were incubated with horseradish peroxidase–conjugated secondary antibody (1:1,000; Cell Signaling Technology) for 2 h at room temperature. Immunoreactive protein bands were visualized by chemiluminescence using a Syngene Bio Imaging Device (Syngene, Cambridge, UK). Densitometric analysis of immunoblots was performed using ImageJ software (National Institutes of Health, Bethesda, MD, USA).

### Histopathological assays

Mice were sacrificed and their hearts harvested and fixed in 4% paraformaldehyde, embedded in paraffin and sectioned (3–4 μm thickness) [14]. After dehydration, sections were stained with hematoxylin-eosin (H-E) and prepared for Masson's trichrome staining. All images were obtained using a light microscope (original magnification 400×; Nikon, Tokyo, Japan). Cell apoptosis rates in the myocardium were determined by terminal deoxynucleotidyl transferase dUTP nick end labeling (TUNEL) assay, using the TUNEL assay kit (Roche Applied Science, Indianapolis, IN, USA). according to the manufacturer’s instructions. Apoptotic, or TUNEL-positive, cells were counted under a light microscope (original magnification 400×).

### Statistical analysis

All data are expressed as means ± standard deviation (SD) and were analyzed by SPSS 10.0 software. Statistical differences among groups were determined by the multiple-comparison one-way ANOVA with Bonferroni corrections. Differences with *p* < 0.05 were considered statistically significant.

## Results

### Treatment with HG induced apoptosis in cardiomyocytes by activation of the IKK-IκBα-NF-κB pathway

**Fig 1:**
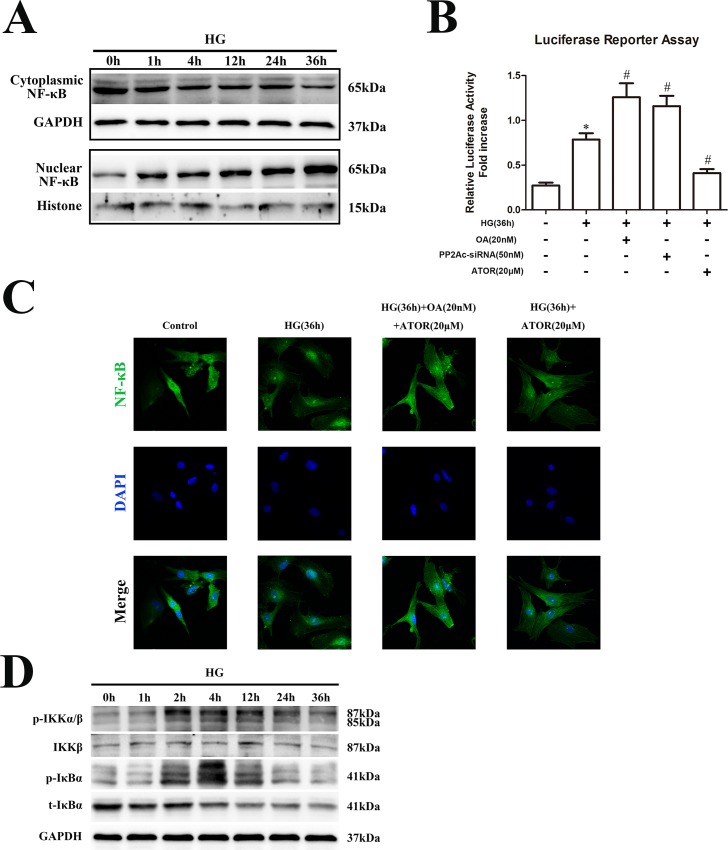
HG treatment induced apoptosis in cardiomyocytes by activation of the IKK-IκBα-NF-κB pathway. **(A).** Primary cultures of neonatal rat cardiomyocytes were exposed to HG for different time periods (0, 1, 4, 12, 24 or 36 h), then cytoplasmic and nuclear proteins were extracted and nuclear translocation of NF-κB detected by western blotting. **(B).** H9C2 cardiomyocytes were exposed to HG for 36 h, in the presence of OA, PP2Ac-siRNA or ATOR, and NF-κB luciferase reporter assays were performed. **(C).** Neonatal rat cardiomyocytes were pretreated with okadaic acid (20 nM) for 1 h, treated with ATOR (20 μM) for 30 min, then exposed to HG for an additional 36 h. The immunofluorescence assay was performed to determine nuclear translocation of NF-κB. **(D).** Neonatal rat cardiomyocytes were exposed to HG for different time periods (0, 1, 2, 4, 12, 24 or 36 h) and total proteins were extracted. Phospho-IKK (p-IKK), p-IκBα, total IKK (t-IKK) and t-IκBα were detected by western blotting.

Sustained activation of NF-κB signaling in HG-treated cardiomyocytes was previously reported [10]. To verify effects of HG on NF-κB activation, primary cultures of neonatal rat cardiomyocytes were exposed to HG (33 mM) for various periods and nuclear translocation of NF-κB analyzed by western blotting. As shown in Fig 1A and S1A Fig, nuclear translocation of NF-κB was observed after 1 h of HG treatment and peaked at 36 h. Moreover, to confirm effects of HG on NF-κB promoter activity, H9C2 cardiomyocytes were transfected with a construct containing the NF-κB-responsive luciferase reporter gene (NF-κB-Luc) and luciferase activity measured after treating cells with HG for 36 h. Luciferase activity was increased after 36 h HG treatment (Fig 1B). Next, we evaluated the degree of nuclear translocation of NF-κB in cardiomyocytes, after exposure to HG for 36 h, using immunofluorescence. As shown in Fig 1C and S1B Fig, nuclear translocation of NF-κB was significantly increased by HG treatment of neonatal rat cardiomyocytes.

Next, we determined effects of HG on IKK/IκBα activation. Neonatal rat cardiomyocytes were exposed to HG for 0, 1, 2, 4, 12, 24 or 36 h, then levels of phosphorylated (phos)-IKK and phos-IκBα were measured. As shown in Fig 1D and S1C Fig, IKK/IκBα was phosphorylated, that is, activated, in a sustained fashion in cells exposed to HG, whereas IKK/ IκBα phosphorylation peaked at 4 h. These data demonstrated that HG induced a sustained activation of IKK/IκBα and subsequent NF-κB nuclear translocation in cardiomyocytes. In addition to its phosphorylation, the sustained degradation of IκBα, by the ubiquitin-proteasome pathway, may be involved.

**Fig 2:**
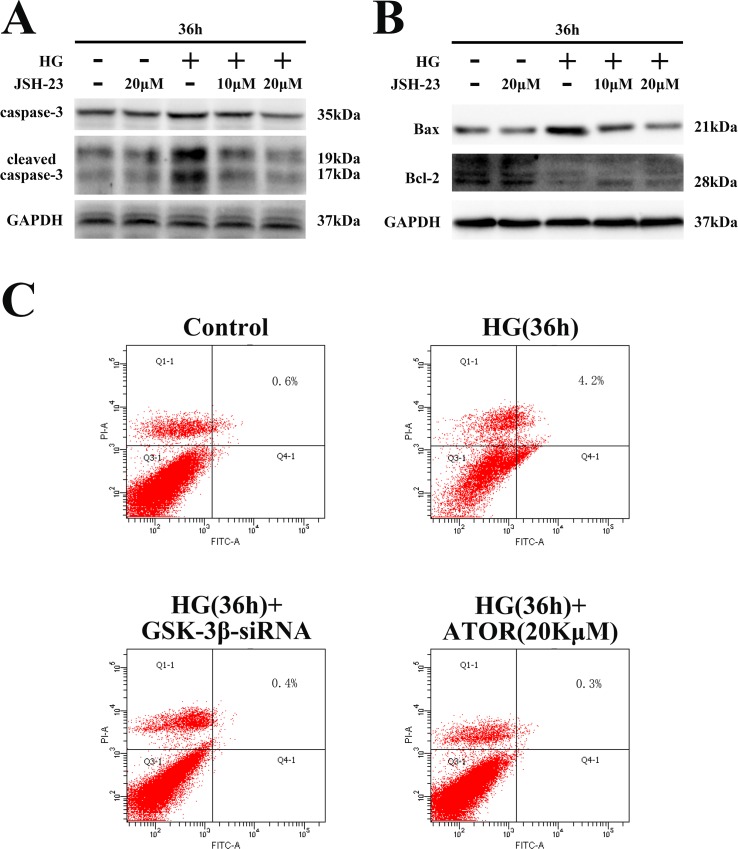
HG-induced apoptosis in neonatal rat cardiomyocytes was mediated by activation of the NF-κB pathway. **(A-B).** Neonatal rat cardiomyocytes were pretreated with 10–20 μM JSH-23 and exposed to HG for 36 h. Levels of caspase-3 and cleaved caspase-3 and Bax/Bcl-2 ratios were determined by western blotting. **(C).** Apoptotic cells stained with Annexin V-FITC and PI were detected by flow cytometry. In the 2D coordinates, the X-axis shows intensity of the FITC signal and the Y-axis shows that of the PI signal.

Apoptosis was significantly increased in cardiomyocytes exposed to HG. As shown in Fig 2A and 2B and S1D Fig, apoptosis markers, caspase-3, cleaved caspase-3 and the ratio of Bax to Bcl-2 (Bax/Bcl-2), were significantly increased in neonatal cardiomyocytes after HG treatment for 36 h. Flow cytometry results indicated increased apoptosis in cardiomyocytes exposed to HG (Fig 2C). To confirm that NF-κB was involved in HG-induced cardiomyocyte apoptosis, we used JSH-23 (MedChem Express, New Jersey, NJ, USA), a cell-permeable diamino compound that selectively blocks nuclear translocation of NF-κB without affecting IκB degradation. As shown in Fig 2A and 2B and S1D Fig, levels of caspase-3 and cleaved caspase-3 and the Bax/Bcl-2 ratio were decreased, in a dose-dependent manner, in cells pretreated with JSH-23. These results indicated that sustained activation of NF-κB was involved in HG-induced apoptosis in neonatal cardiomyocytes.

### Effects of PP2Ac on HG-induced sustained phosphorylation of IKK/IκBα in H9C2 cells

**Fig 3:**
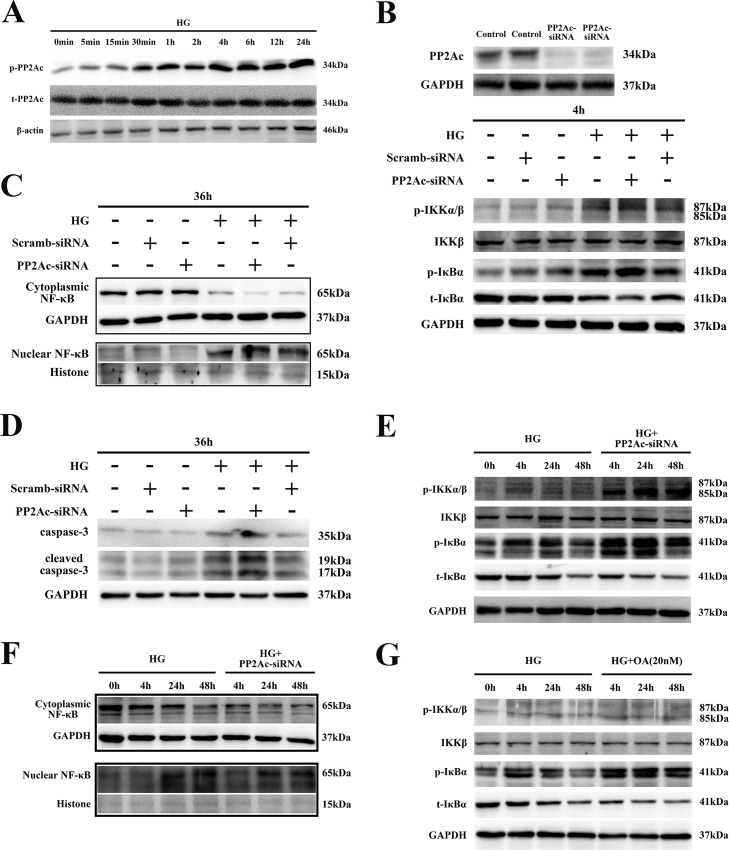
Effects of PP2Ac on HG-induced sustained phosphorylation of IKK/IκBα in H9C2cells. **(A).** H9C2 cells were exposed to HG for different time periods (0, 5, 15 or 30 min; or 1, 2, 4, 6, 12 or 24 h), total proteins were extracted and PP2Ac phosphorylation detected by western blotting. **(B).** After transfection with PP2Ac-siRNA for 48 h, cells were exposed to HG for an additional 4 h and IKK/IκBα was detected by western blotting. **(C).** After transfection with PP2Ac-siRNA for 24 h, cells were exposed to HG for 36 h. Then, cytoplasmic and nuclear proteins were extracted and NF-κB detected by western blotting. **(D).** Total protein was extracted after transfecting cells with PP2Ac-siRNA and exposing them to HG for 36 h. Apoptotic proteins were detected by western blotting. **(E).** After H9C2 cells were transfected with or without PP2Ac-siRNA, then exposed to HG for different time periods (4, 24 or 48 h), IKK/IκBα expression and phosphorylation and nuclear translocation of NF-κB were **(F)** detected by western blotting. **(G).** H9C2 cells were exposed to HG for 4, 24 or 48 h, after adding OA, and IKK/IκBα expression and phosphorylation were determined by western blotting.

PP2A was reported to play an important role in regulating phosphorylation of IKK and IκBα in cardiomyocytes [10]. To further elucidate the association between PP2A and the IKK-IκBα-NF-κB signaling pathway in cardiomyocytes during HG treatment, H9C2 cells were exposed to HG for various time periods and tyrosine phosphorylation of PP2A (that is, its inactivation) was measured. As shown in Fig 3A and S2A Fig, PP2A phosphorylation was rapidly increased within 5 min of HG exposure and was maintained at a high level for 24 h. This indicated that PP2A activity was continuously inhibited in cardiomyocytes by HG treatment. Next, PP2Ac expression was silenced by transfecting H9C2 cells with siRNA. As shown in Fig 3B and S2B Fig, IKK/IκBα phosphorylation and IκBα degradation in transfected cells were further enhanced after HG-exposure for 4 h. Moreover, nuclear translocation of NF-κB was also further increased in cells transfected with PP2Ac-siRNA (Fig 3C and S2C Fig). Luciferase activity, indicating transcriptionally active NF-κB, was further increased in cells transfected with PP2Ac-siRNA or in those treated with OA, a selective PP2A inhibitor (Fig 1B). We also observed that, after inhibition of PP2Ac expression, cell apoptosis was markedly induced, as shown by increased levels of the apoptotic proteins caspase-3 and cleaved caspase-3 (Fig 3D and S2D Fig). In addition, in cells transfected with siRNA, HG exposure for 4, 24 or 48 h induced a sustained phosphorylation of IKK and IκBα as well as nuclear translocation of NF-κB (Fig 3E and 3F, S2E and S2F Fig). Furthermore, pretreatment with OA for 4–48 h before HG exposure also enhanced IKK/IκBα phosphorylation (Fig 3G and S2G Fig). These data further confirmed that PP2A could negatively regulate the HG-induced IKK-IκBα-NF-κB signaling pathway in H9C2 cardiomyocytes.

### Role of GSK-3β in HG-induced sustained phosphorylation of PP2Ac-IKK-IκBα in H9C2 cells

**Fig 4:**
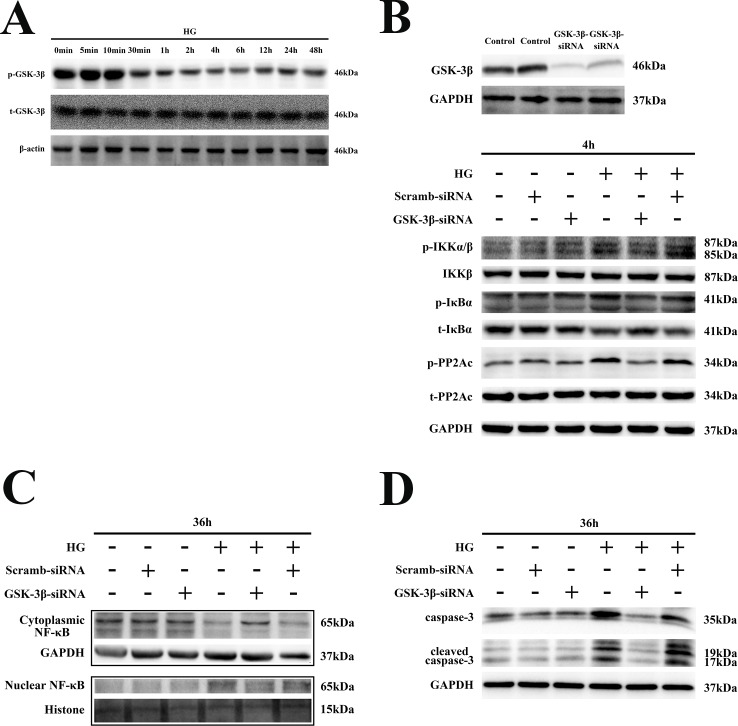
Role of GSK-3β in HG-induced sustained phosphorylation of PP2Ac-IKK-IκBα in H9C2 cells. **(A).** H9C2 cardiomyocytes were exposed to HG for 0, 5, 15 or 30 min; or 1, 2, 4, 6, 12, 24 or 48 h, then total protein was extracted and GSK-3β phosphorylation detected by western blotting. **(B).** After transfection with GSK-3β-siRNA for 48 h, cells were exposed to HG for an additional 4 h and phosphorylation of PP2Ac and IKK/IκBα detected by western blotting. **(C).** After transfection with GSK-3β-siRNA for 24 h, cells were exposed to HG for another 36 h. Then, cytoplasmic and nuclear proteins were extracted and NF-κB detected by western blotting. **(D).** Total protein was extracted after transfecting cells with GSK-3β-siRNA and exposing them to HG for 36 h. Apoptotic proteins were detected by western blotting.

To verify whether GSK-3β inhibition decreased PP2Ac phosphorylation in cardiomyocytes exposed to HG, we determined phosphorylation levels of PP2Ac, IKK and IκBα by silencing GSK-3β expression. As shown in Fig 4A and S3A Fig, sustained GSK-3β activation was observed in H9C2 cells, with GSK-3β being significantly dephosphorylated during 4–6 h of HG exposure. As shown in Fig 4B and S3B Fig, HG-induced phosphorylation of PP2Ac and IKK and phosphorylation and degradation of IκBα were markedly inhibited in cells transfected with GSK-3β siRNA. Furthermore, nuclear translocation of NF-κB and expression of apoptotic proteins were also inhibited in cells transfected with GSK-3β siRNA (Fig 4C and 4D, S3C and S3D Fig). Moreover, in H9C2 cells transfected with GSK-3β siRNA, apoptosis was markedly decreased, as assessed by flow cytometry (Fig 2C). These results indicated that GSK-3β played an important role in regulating the PP2A-mediated sustained activation of the IKK-IκB-NF-κB signaling pathway in HG-stimulated H9C2 cells.

### Effects of ATOR on HG-induced activation of the GSK-3β-PP2Ac-NF-κB pathway in H9C2 cells

**Fig 5:**
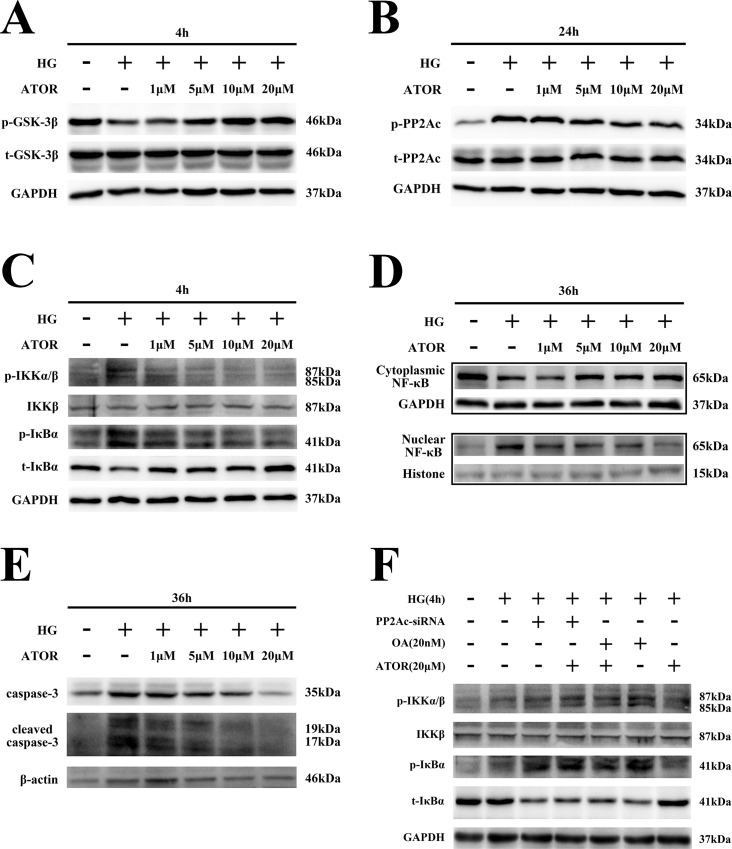
Effects of ATOR on HG-induced activation of the GSK-3β-PP2Ac-IKK-IκBα-NF-κB pathway in H9C2 cells. **(A).** H9C2 cells were pretreated with ATOR at different concentrations (1, 5, 10 or 20 μM) then exposed to HG for 4 h and proteins extracted. GSK-3β phosphorylation was detected by western blotting. **(B).** H9C2 cells were pretreated with ATOR at different concentrations then exposed to HG for 24 h and phosphorylation of PP2Ac detected by western blotting. **(C).** H9C2 cells were pretreated with ATOR at different concentrations then exposed to HG for 4 h and proteins extracted. IKK/IκBα phosphorylation was detected by western blotting. **(D).** H9C2 cardiomyocytes were pretreated with ATOR at different concentrations and exposed to HG for 36 h, then cytoplasmic and nuclear proteins extracted. Nuclear translocation of NF-κB was determined by western blotting. **(E).** Cells were treated as described for Fig 5D, total proteins were extracted and apoptosis-associated proteins detected by western blotting. **(F).** H9C2 cells were pretreated with OA, OA+ATOR, PP2Ac-siRNA+ATOR or PP2Ac-siRNA before exposure to HG for 4 h. IKK and IκBα expression was detected by western blotting.

To elucidate effects of ATOR on HG-induced apoptosis and activation of NF-κB signaling, H9C2 cells were pretreated with ATOR at various concentrations (1, 5, 10 or 20 μM) and then exposed to HG for 4 or 36 h. As shown in Fig 5A and S4A Fig, GSK-3β phosphorylation was markedly increased, in a dose-dependent manner, by ATOR treatment. PP2Ac phosphorylation was lower in cardiomyocytes pretreated with ATOR, as compared with in the HG group (Fig 5B and S4B Fig). Moreover, ATOR appeared to inhibit IKK/IκBα activation and prevent IκBα degradation in a dose-dependent manner (Fig 5C and S4C Fig). In cells pretreated with ATOR, nuclear translocation was also attenuated (Fig 5D and S4D Fig), which was further confirmed by immunofluorescence (Fig 1C and S1B Fig). Results of the luciferase reporter assay also showed that ATOR suppressed transactivation of NF-κB in HG-treated H9C2 cells (Fig 1B). Next, we measured the effect of ATOR on apoptosis in H9C2 cells by western blotting. Levels of the apoptotic proteins caspase-3 and cleaved caspase-3 were significantly decreased in cells pretreated with ATOR and this effect was dose-dependent (Fig 5E and S4E Fig), consistent with results obtained by flow cytometry (Fig 2C). These data demonstrated that ATOR inhibited apoptosis in H9C2 cardiomyocytes undergoing HG exposure, through suppressing activation of the GSK-3β-PP2Ac-IKK-IκBα-NF-κB pathway.

### The role of PP2Ac in ATOR-mediated inhibition of IKK/IκBα in H9C2 cardiomyocytes

We found that ATOR attenuated PP2Ac phosphorylation at Tyr-307 in H9C2 cells exposed to HG (Fig 5B). To further demonstrate that effects of ATOR on IKK/IκB were mediated by PP2Ac, cells were pretreated with OA or PP2Ac-siRNA, with or without ATOR (20μM), prior to HG exposure. As shown in Fig 5F and S4F Fig, IKK/IκBα phosphorylation was enhanced in cells transfected with PP2Ac-siRNA or pretreated with OA, as compared with control cells, after exposure to HG for 4 h. However, in cells pretreated with OA or PP2Ac-siRNA, ATOR did not inhibit IKK/IκBα phosphorylation. These results further indicated that PP2Ac was involved in the ATOR-mediated inhibition of IKK/IκBα phosphorylation.

### Effects of ATOR on body weight, heart weight, tibial length, blood glucose and blood lipid levels in diabetic animals

As shown in Table 1, in the normal group, ATOR treatment did not affect body weight (BW), heart weight (HW), tibial length (TL) or the ratios HW/BW and HW/TL. Compared with the normal group, treatment with or without ATOR and diabetic mice treatment with ATOR, diabetic mice demonstrated lower body weight, heart weight and HW/TL and increased HW/BW. However, TL did not differ in the diabetic and non-diabetic groups. Next, the blood glucose and lipids levels were determined in normal and diabetic mice, each with or without ATOR treatment. Compared with untreated diabetic mice, the other three groups exhibited significantly lower blood glucose, total cholesterol, triacylglycerol and LDL-cholesterol levels, as well as significantly increased HDL-cholesterol levels.

**Table 1:** Biometric parameters of experimental mice at 12 wk.

Parameters	Control	Control+ATOR	DM	DM+ ATOR
Body weight (g)	30.6 ± 1.3*	30.7 ±0.8*	23.9 ±1.8	26.5 ±1.1*
Heart weight (mg)	130.8 ±4.16*	131.2 ± 6.28*	122.5 ± 7.13	129.3 ± 4.86*
Heart/body weight ratio (mg/g)	4.30 ± 0.53*	4.28 ± 0.78*	5.22 ± 0.48	4.50 ± 0.33*
Tibial length (mm)	18.1 ± 0.26	18.1 ± 0.18	17.9 ± 0.23	18.0 ± 0.13
Heart weight/Tibial length (mg/mm)	7.22 ± 0.34*	7.24 ± 0.28*	6.80 ± 0.11	7.10 ± 0.13*
Blood glucose (mmol/L)	6.78 ± 0.27*	6.70 ± 0.22*	22.65 ± 4.06	22.11 ± 3.08
Total cholesterol (mmol/l)	2.7± 0.46*	2.67 ± 0.51*	5.86 ± 0.76	3.16 ± 0.66*
HDL-cholesterol (mmol/l)	1.22 ± 0.23*	1.25 ± 0.18*	0.67 ± 0.57	1.07± 0.28*
LDL-cholesterol (mmol/l)	0.65 ± 0.16*	0.64 ± 0.18*	1.31 ± 0.20	0.66 ± 0.13*
Triacylglycerol (mmol/l)	0.96 ± 0.18*	0.94 ± 0.12*	1.61 ± 0.22	1.31 ± 0.12*

control, *n* = 15; Control+ ATOR, *n* = 15; DM, *n* = 12; DM+ ATOR, *n* = 10

*, *p<*0.05 vs. DM group

### Effects of ATOR on cardiomyocyte apoptosis and the NF-κB pathway in diabetic hearts

**Fig 6:**
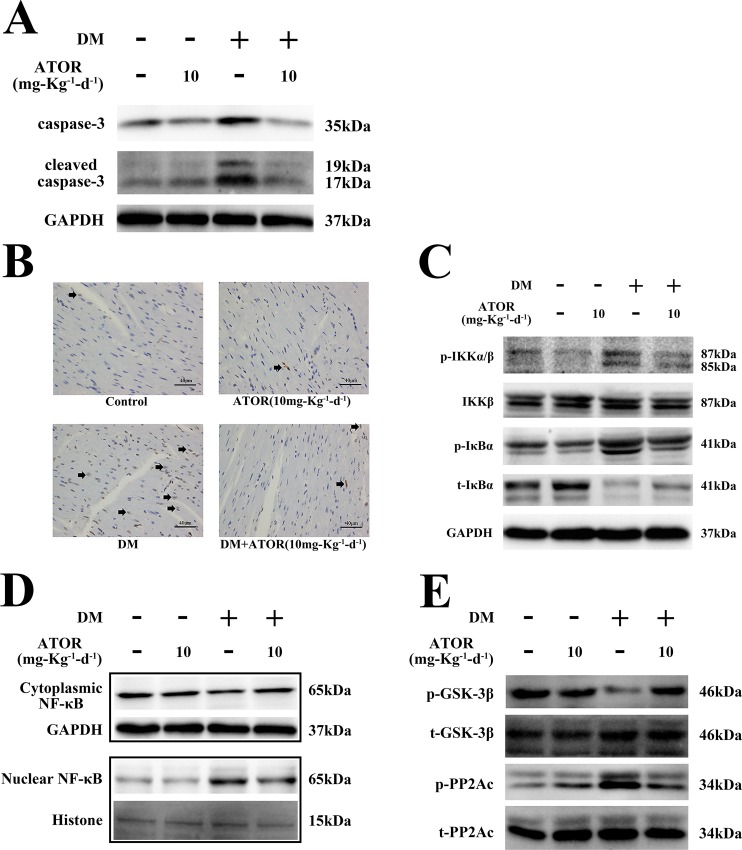
Effects of ATOR on cardiomyocyte apoptosis and the NF-κB pathway in diabetic hearts. **(A).** Total protein extracted from heart tissues was subjected to western blot analysis for caspase-3 and cleaved caspase-3. **(B).** Heart tissues were sectioned at 3–4 μm and the slides were processed for TUNEL staining to detect apoptotic cells (arrows represent TUNEL-positive cells). **(C).** Total protein was extracted from heart tissues and IKK/IκBα expression detected by western blotting. **(D).** Cytoplasmic and nuclear proteins were extracted from heart tissues and nuclear translocation of NF-κB determined. **(E).** GSK-3β and PP2Ac phosphorylation was detected in total protein extracts from heart tissues. TUNEL assay images were obtained by microscopy with original magnification 400×.

In hearts from the DM, there was a marked increase in caspase-3 and cleaved caspase-3 (Fig 6A and S5A Fig), effects confirmed by an increase in TUNEL-positive cells (Fig 6B and S5B Fig). However, treatment of DM with ATOR significantly prevented the increases in caspase-3 and cleaved caspase-3 and induction of TUNEL-positive cells (Fig 6A and 6B, S5A and S5B Fig). We also examined activation of the NF-κB pathway in diabetic hearts. As shown in Fig 6C and S5C Fig, markedly increased IKK/IκBα phosphorylation was observed in diabetic hearts, and this was accompanied by IκBα degradation. Nuclear translocation of NF-κB was also observed in the hearts from DM (Fig 6D and S5D Fig). However, IKK/IκBα phosphorylation, IκBα degradation and nuclear translocation of NF-κB were significant inhibited by ATOR treatment (Fig 6C and 6D, S5C and S5D Fig). In addition, tyrosine phosphorylation of PP2Ac and GSK-3β activation were both also inhibited by ATOR treatment, compared with in untreated DM (Fig 6E and S5E Fig). These data suggested that the effects of ATOR on apoptosis in diabetic hearts were mediated by the GSK-3β-PP2Ac-IKK-IκBα-NF-κB pathway, confirming our *in vitro* findings.

### Effects of ATOR on diabetes-induced alterations in histology and cardiac function

**Fig 7:**
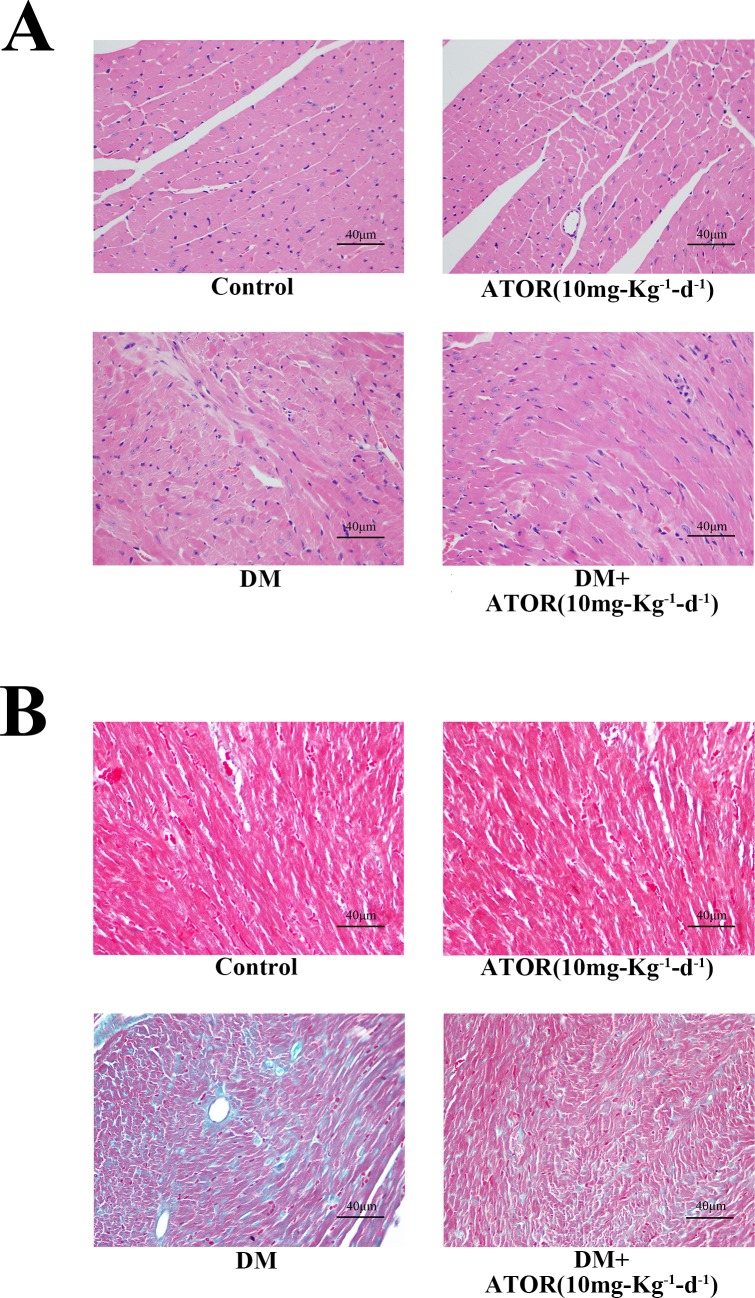
Effects of ATOR on diabetes-induced histological alterations and cardiac dysfunction. **(A).** H-E staining for heart tissues in each group. **(B).** Masson's trichrome staining for heart tissues in each group. Data are means ± SD from three independent experiments. *, *p*<0.05 vs control; #, *p*<0.05 vs HG. H-E staining and Masson's trichrome staining images were obtained by microscopy with original magnification 400×.

**Fig 8:**
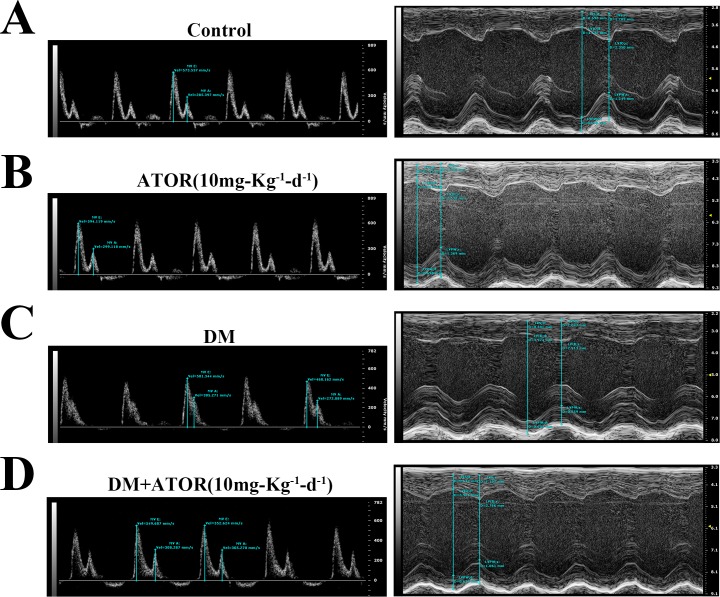
Effects of ATOR on diabetes-induced alterations in cardiac function. Doppler echocardiographic images obtained from hearts of normal mice **(A)**, normal mice treated with ATOR **(B)**, diabetic mice **(C)** and diabetic mice treated with ATOR **(D)** for 12 wk. E, peak velocity of the early ventricular filling (E wave); A, peak velocity of the late ventricular filling (A wave).

We examined fibrosis and histopathology in the diabetic hearts. As shown in Fig 7A and 7B and S5F Fig, diabetic hearts had broken fibers, deranged cellular structures, diffuse disruption of the cardiomyocytes and collagen accumulation, shown by H-E and Masson's trichrome staining, and these effects were partially improved in mice treated with ATOR. The cardiac pathological changes in DM seemed likely to have resulted in diabetes-induced cardiac dysfunction. We therefore examined the cardiac function of all mice by transthoracic echocardiography (Fig 8A–8D). Diabetes markedly deteriorated the function of diastolic and systolic LV, and this was largely attenuated in mice treated with ATOR for 12 weeks (Table 2).

**Table 2:** Transthoracic echocardiographic parameters of DM with type 1 diabetes for 12 weeks.

Parameters	Control	Control+ATOR	DM	DM + ATOR
HR (bpm)	568.6 ± 14.507	566.5 ± 10.108	593.9 ± 22.822	587.5 ± 24.023
IVS, d (mm)	0.72 ± 0.028*	0.72 ± 0.043*	0.64 ± 0.042	0.69 ± 0.050*
IVS, s (mm)	1.25 ± 0.063*	1.27 ± 0.078*	1.01 ± 0.052	1.11 ± 0.061*
LVID, d (mm)	3.86 ± 0.156	3.86 ± 0.118	3.90 ± 0.125	3.88 ± 0.132
LVID, s (mm)	2.35 ± 0.128*	2.34 ± 0.073*	2.86 ± 0.117	2.60 ± 0.203*
LVPW, d (mm)	0.72 ± 0.069*	0.72 ± 0.047*	0.65 ± 0.060	0.76 ± 0.084*
LVPW, s (mm)	1.26 ± 0.078*	1.28 ± 0.061*	1.02 ± 0.135	1.18 ± 0.090*
EF%	69.44 ± 1.842*	70.20 ± 1.502*	58.86 ± 3.422	65.64 ± 2.659*
FS%	45.69 ± 2.353*	46.07 ± 3.618*	31.05 ± 2.571	36.85 ± 4.295*
E/A ratio	1.95 ± 0.112*	1.97 ± 0.072*	1.62 ± 0.090	1.80 ± 0.236*
LV mass (mg)	90.67 ± 5.679*	90.88 ± 4.552*	80.30 ± 4.550	87.11 ± 6.424*
LV mass, Co (mg)	73.33 ± 5.106*	73.76 ± 3.878*	65.84 ± 2.606	70.69 ± 4.215*
LV vol, d (μl)	60.73 ± 1.561	60.02 ± 0.763	61.73 ± 5.432	60.77 ± 7.000
LV vol, s (μl)	18.55 ± 1.499*	18.17 ± 1.121*	25.65 ± 4.809	22.33 ± 2.149*

control, *n* = 15; Control+ ATOR, *n* = 15; DM, *n* = 12; DM+ ATOR, *n* = 10

*, *p<*0.05 vs. DM group

## Discussion

The major findings of our study were: 1) Persistent inactivation of PP2Ac, caused by activated GSK-3β, in HG-treated cardiomyocytes resulted in sustained activation of the NF-кB signaling pathway, initiating apoptosis. 2) IKK/IкBα phosphorylation and NF-кB nuclear translocation in HG-treated cardiomyocytes were markedly inhibited and apoptosis was attenuated by ATOR treatment, through GSK-3β inactivation and subsequent PP2Ac activation. These effects were abolished by treatment with OA or by silencing PP2Ac expression. 3) GSK-3β activation, IKK/IкBα phosphorylation and NF-кB nuclear translocation, but not PP2Ac activation, were significantly suppressed in DM treated with ATOR. Histological abnormalities, fibrosis, apoptosis and cardiac dysfunction were also improved by ATOR administration in DM.

Hyperglycemia, which characterizes type 1 and 2 diabetes mellitus, is an independent risk factor for development of DCM [20]. Previous studies showed that attenuation of HG-induced apoptosis improved cardiac function and prevented DCM [20]. Others reported that chronic activation of NF-κB signaling resulted in increased apoptotic cell death and progression toward heart failure [21]. Thus, targeted inhibition of persistent activation of NF-κB signaling might effectively treat DCM. We found that IKK phosphorylation and subsequent phosphorylation of IκBα and nuclear translocation of NF-κB, contributing to caspase-3 activation, were sustained in cardiomyocytes exposed to HG. These findings were consistent with earlier studies [10, 16]. We also found that the sustained activation of NF-κB signaling and caspase-3 activation were inhibited in a concentration-dependent manner by ATOR in cardiomyocytes cultured in HG. This was consistent with the histological and other cardiac improvements in DM given ATOR. Therefore, the anti-apoptotic and cardioprotective effects of ATOR indicated that it is a potentially useful agent for treating DCM, through its inhibition of the sustained activation of NF-κB signaling.

ATOR is an inhibitor of HMG-CoA reductase and is used as a cholesterol-lowering medication. In recent studies, the drug alleviated progression of experimental DCM, possibly by a protective effect against apoptosis, independent of its LDL-cholesterol-lowering properties [14]. We demonstrated in this study that the anti-apoptotic and cardioprotective effects of ATOR may be explained by inhibition of sustained activation of NF-κB signaling. However, the molecular mechanisms through which ATOR can negatively regulate NF-κB signaling remain unclear. Inactive NF-κB is primarily located in the cytoplasm, associated with the inhibitor IκB. Among the IκB protein family members, IκBα is the most extensively studied, primarily because its degradation is significantly faster than that of others in most canonical pathways. Phosphorylation and degradation of IκBα are initiated by upstream activation of IKK, which contains two catalytic subunits, IKKα and IKKβ and a noncatalytic regulatory subunit, IKKγ/NEMO (NF-κB essential modulator). During activation of NF-κB signaling pathways, IκBα is rapidly degraded, which leads to release of NF-κB and its nuclear localization. After activation, NF-κB binds to resynthesized IκB, leading to termination of NF-κB-dependent transcription. This feedback loop is important to maintain normal cellular responsiveness mediated by NF-κB [22, 23]. Nizamutdinova *et al* [10] reported that continuous PP2Ac phosphorylation at Tyr307 (that is, its inactivation) led to dysregulated IKK or IκB, initiating sustained NF-κB nuclear translocation in HG-cultured cardiomyocytes. These investigators also found that enhancing PP2Ac activity inhibited the persistent activation of NF-κB signaling, resulting in protection of cells from HG-induced apoptosis. Thus, PP2Ac plays an important role in the pathogenesis of DCM. It is interesting to note that PP2A is a substrate for GSK-3β in multiple cell types, such as HEK293 cells and N2a cells, and that activated GSK-3β can negatively regulate PP2Ac activity [11, 12]. Henriksen *et al* [13] reported that GSK-3β activity was increased in both diabetes and insulin resistance. Therefore, activated GSK-3β might lead to PP2Ac inactivation and, subsequently, to sustained activation of NF-κB signaling. Indeed, we found that IKK/IкBα phosphorylation and NF-кB nuclear translocation were decreased in cells in which expression of GSK-3β had been silenced. This resulted in decreased PP2Ac activation, compared with in control cells, in response to HG exposure. It was reported that ATOR enhanced neurite outgrowth in cultured cortical neurons via inactivating GSK-3β [15]. Thus, the anti-apoptotic and cardioprotective effects of ATOR in HG-treated cardiomyocytes and in an experimental model of DCM may depend upon GSK-3β inactivation and subsequent PP2Ac activation, as well as on suppression of the constitutive activation of the NF-κB signaling pathway. In our study, GSK-3β activity and the sustained activation of NF-κB signaling were significantly suppressed, while PP2Ac activation was restored, both in HG-cultured cardiomyocytes and in DM treated with ATOR. Histological abnormalities, fibrosis, apoptosis and cardiac dysfunction were improved in DM treated with ATOR. These findings suggested a mechanism for partial alleviation of experimental DCM progression by ATOR. Furthermore, the anti-apoptotic mechanism of ATOR may partially explain its therapeutic effects in some diseases, including ischemic stroke and Alzheimer’s Disease, which are both associated with apoptosis [24–26] and are effectively treated with ATOR [27, 28]. Previous studies reported that NF-κB activation was indirectly regulated by GSK-3β [23] and that NF-κB activity was inhibited by ATOR in a variety of cell types [29] and animal models [30, 31], and our study further supports the negative regulation of NF-κB by ATOR.

In summary, anti-apoptotic and cardioprotective effects of ATOR in HG-treated cardiomyocytes and in DCM were partially dependent on regulating GSK-3β-PP2Ac-NF-κB signaling. These findings may be of great relevance to other diabetic complications. HG-mediated apoptotic cell death is relevant to such diabetic complications as neuropathy, nephropathy and cardiovascular disease [32] and, in addition, GSK-3β activity is increased in diabetes. Thus, we propose that ATOR has potential therapeutic value for such complications and that this should be further investigated.

## Supporting Information

S1 Fig Related to Figs 1 and 2. **The pro-apoptotic effect of HG treatment on cardiomyocytes was mediated by the NF-κB signaling pathway. (A).** Densitometric quantification of cytoplasmic and nuclear NF-κB protein bands, normalized to those for GAPDH or histone. **(B).** A quantitative analysis of the relative amount of nuclear NF-κB p65 in neonatal rat cardiomyocytes. **(C).** Densitometric quantification of p-IKKα/β, IKKβ, p-IκBα and total IκBα protein bands, normalized to GAPDH. **(D).** Densitometric quantification of pro-apoptotic proteins (caspase-3, cleaved caspase-3 and Bax/Bcl-2), normalized to GAPDH. All densitometric quantification was performed with Image J. All data are means ± SD and were obtained from at least three independent experiments. *, *p*<0.05 vs control; #, *p*<0.05 vs HG.
(TIF)

S2 Fig Related to Fig 3. **Effects of PP2Ac on HG-induced sustained phosphorylation of IKK/IκBα in H9C2cells. (A–G).** Densitometric quantification of protein bands shown in Fig 3A–3G. All densitometric quantification was performed using Image J. Data are means ± SD and were obtained from at least three independent experiments. *, *p*<0.05 vs control; #, *p*<0.05 vs HG.
(TIF)

S3 Fig Related to Fig 4. **Role of GSK-3β in HG-induced sustained phosphorylation of PP2Ac-IKK-IκBα in H9C2 cells. (A–D)** Densitometric quantification of protein bands shown in Fig 4A–4D. All densitometric quantification was performed with Image J. Data are means ± SD and were obtained from at least three independent experiments. *, *p*<0.05 vs control; #, *p*<0.05 vs HG.
(TIF)

S4 Fig Related to Fig 5. **Effects of ATOR on HG-induced activation of the GSK-3β-PP2Ac-IKK-IκBα-NF-κB pathway in H9C2 cells. (A–F)** Densitometric quantification of protein bands shown in Fig 5A–5F. All densitometric quantification was performed with Image J. Data are means ± SD and were obtained from at least three independent experiments. *, *p*<0.05 vs control; #, *p*<0.05 vs HG.
(TIF)

S5 Fig Related to Figs 6 and 7. **Effects of ATOR on cardiomyocyte apoptosis, the NF-κB pathway and histological alterations in diabetic hearts. (A), (C–E).** Densitometric quantification of protein bands shown in Fig 6A and 6C–6E. **(B).** Quantification of TUNEL-positive cells in diabetic hearts, shown in Fig 6B. **(F).** Quantification of collagen areas in diabetic hearts, shown in Fig 7B. Data are means ± SD from three independent experiments. *, *p*<0.05 vs control; #, *p*<0.05 vs HG.
(TIF)
