# Supplementary figures and images for: Atorvastatin Alleviates Experimental Diabetic Cardiomyopathy by Regulating the GSK-3β-PP2Ac-NF-κB Signaling Axis
Source: PLoS One. 2016 Nov 16;11(11):e0166740. doi: 10.1371/journal.pone.0166740 (PMC5112957; doi:10.1371/journal.pone.0166740)

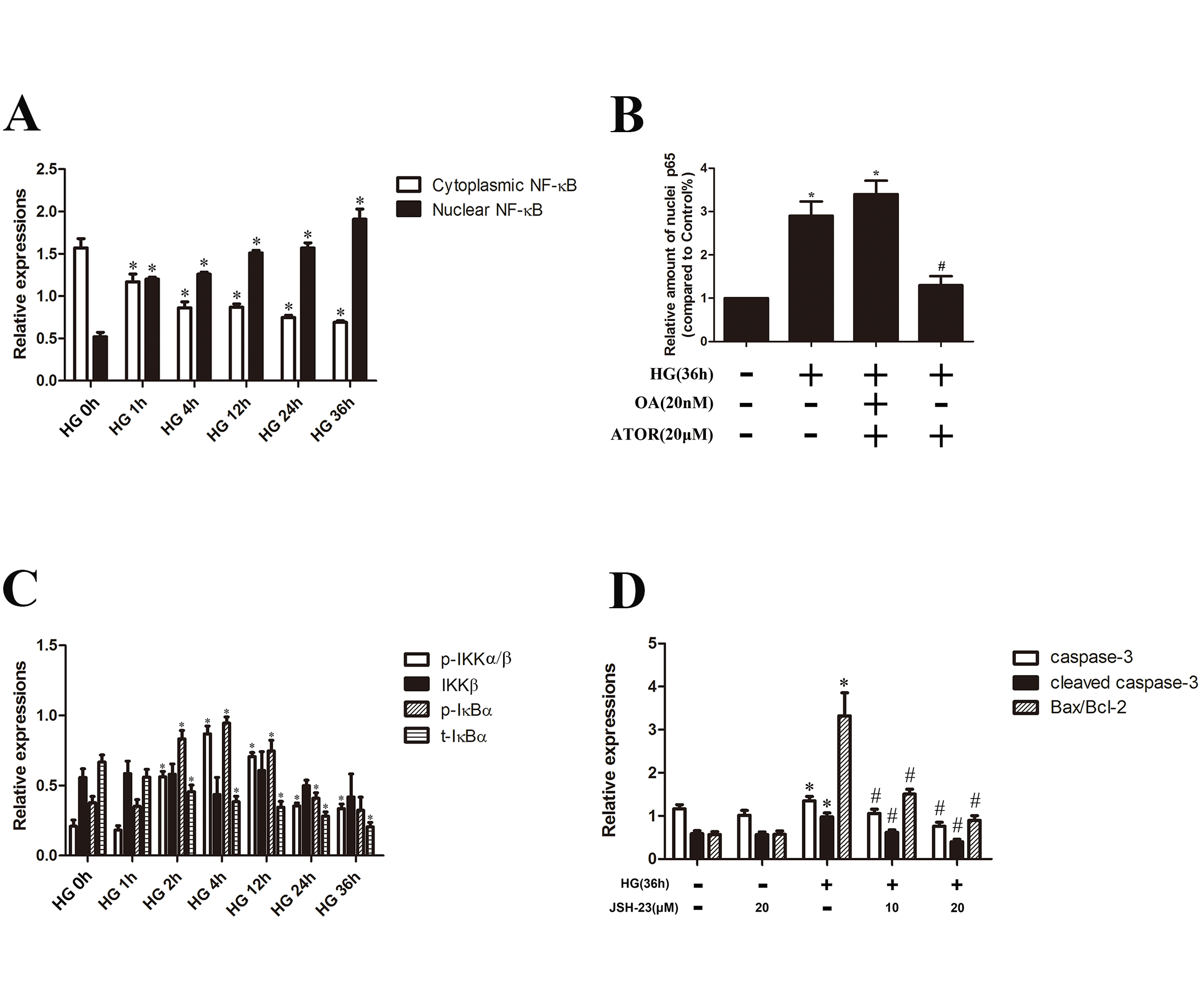

Supplement: S1 Fig — The pro-apoptotic effect of HG treatment on cardiomyocytes was mediated by the NF-κB signaling pathway. (A). Densitometric quantification of cytoplasmic and nuclear NF-κB protein bands, normalized to those for GAPDH or histone. (B). A quantitative analysis of the relative amount of nuclear NF-κB p65 in neonatal rat cardiomyocytes. (C). Densitometric quantification of p-IKKα/β, IKKβ, p-IκBα and total IκBα protein bands, normalized to GAPDH. (D). Densitometric quantification of pro-apoptotic proteins (caspase-3, cleaved caspase-3 and Bax/Bcl-2), normalized to GAPDH. All densitometric quantification was performed with Image J. All data are means ± SD and were obtained from at least three independent experiments. *, p<0.05 vs control; #, p<0.05 vs HG. (TIF) [file pone.0166740.s001.tif]

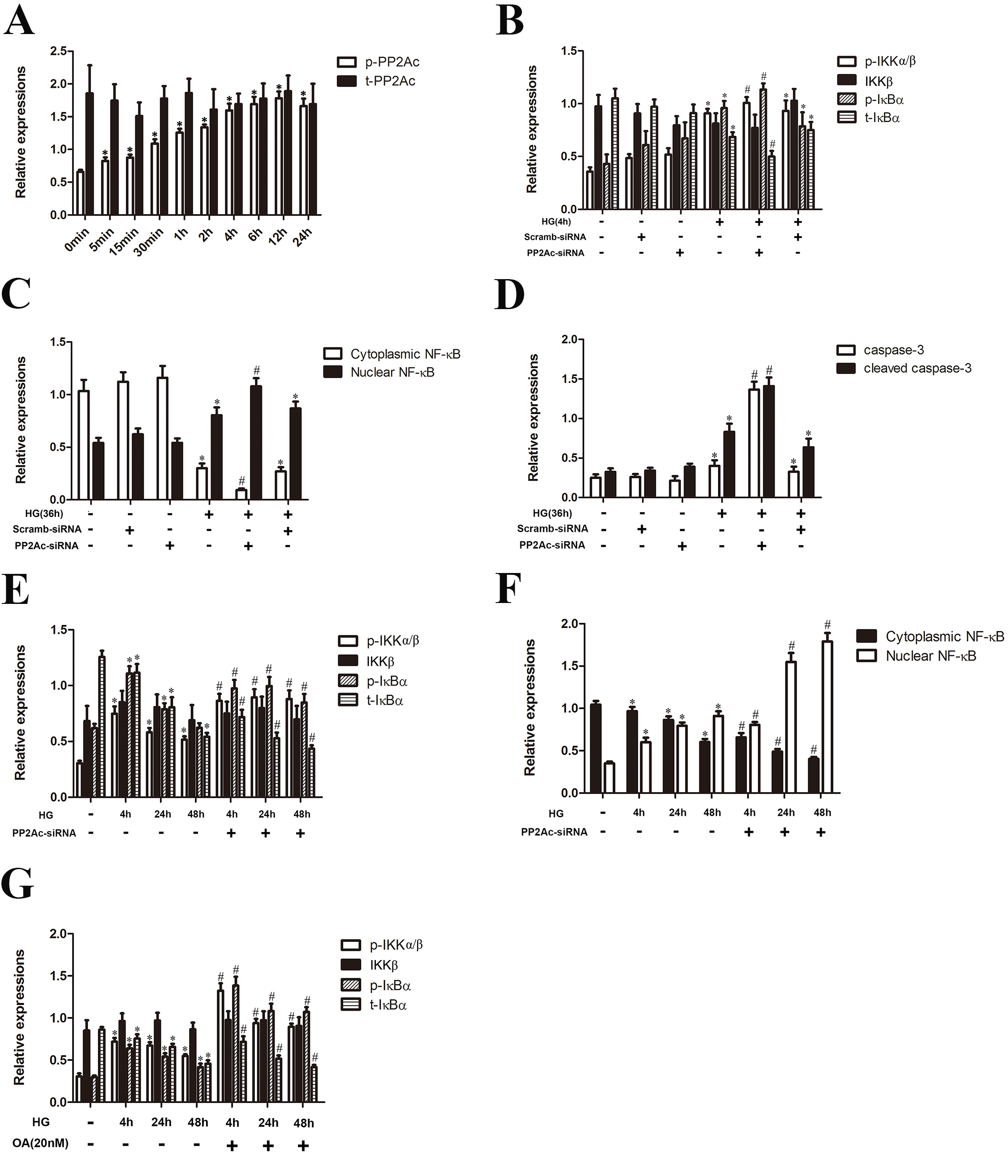

Supplement: S2 Fig — Effects of PP2Ac on HG-induced sustained phosphorylation of IKK/IκBα in H9C2cells. (A–G). Densitometric quantification of protein bands shown in Fig 3A–3G. All densitometric quantification was performed using Image J. Data are means ± SD and were obtained from at least three independent experiments. *, p<0.05 vs control; #, p<0.05 vs HG. (TIF) [file pone.0166740.s002.tif]

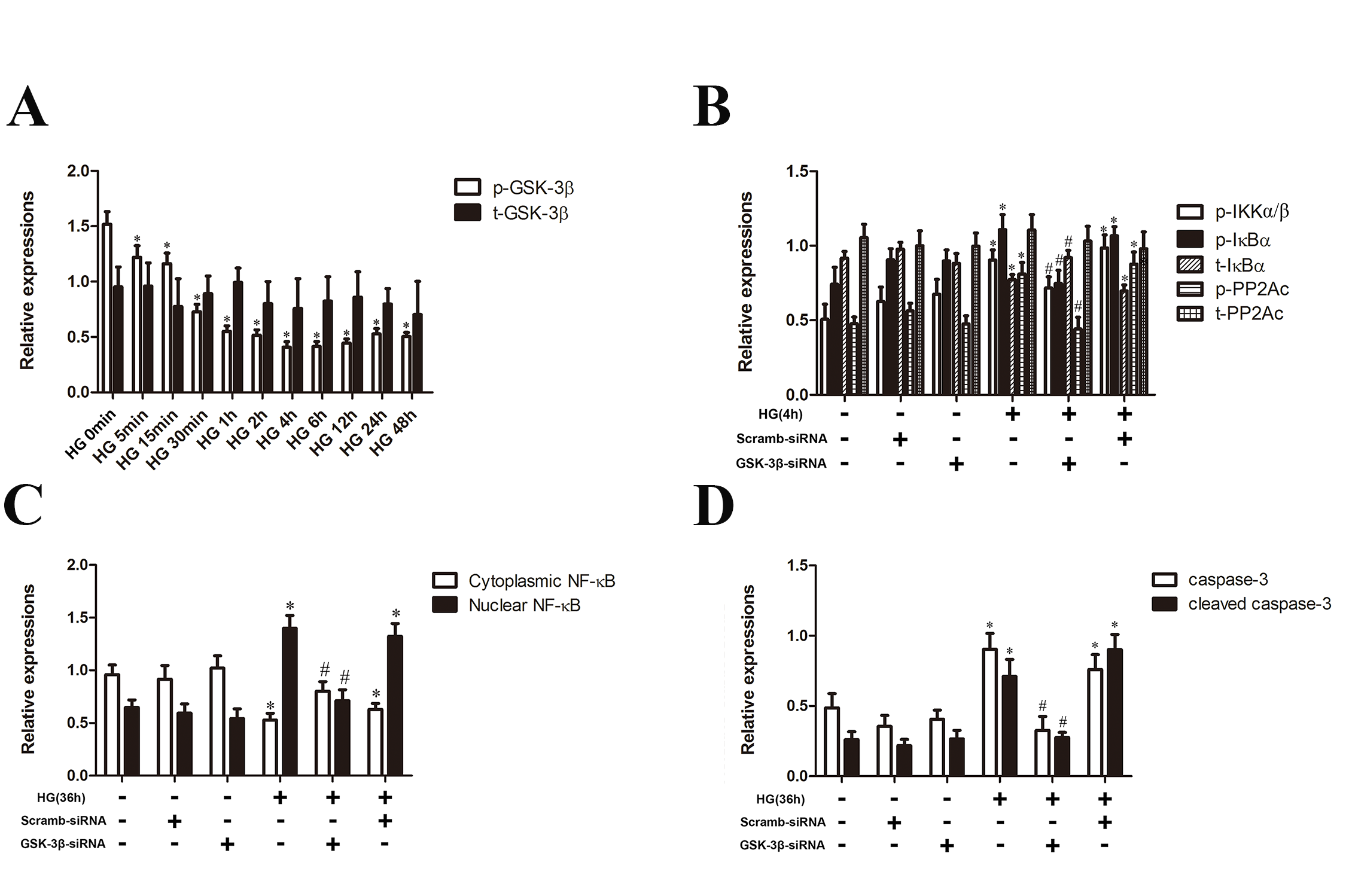

Supplement: S3 Fig — Role of GSK-3β in HG-induced sustained phosphorylation of PP2Ac-IKK-IκBα in H9C2 cells. (A–D) Densitometric quantification of protein bands shown in Fig 4A–4D. All densitometric quantification was performed with Image J. Data are means ± SD and were obtained from at least three independent experiments. *, p<0.05 vs control; #, p<0.05 vs HG. (TIF) [file pone.0166740.s003.tif]

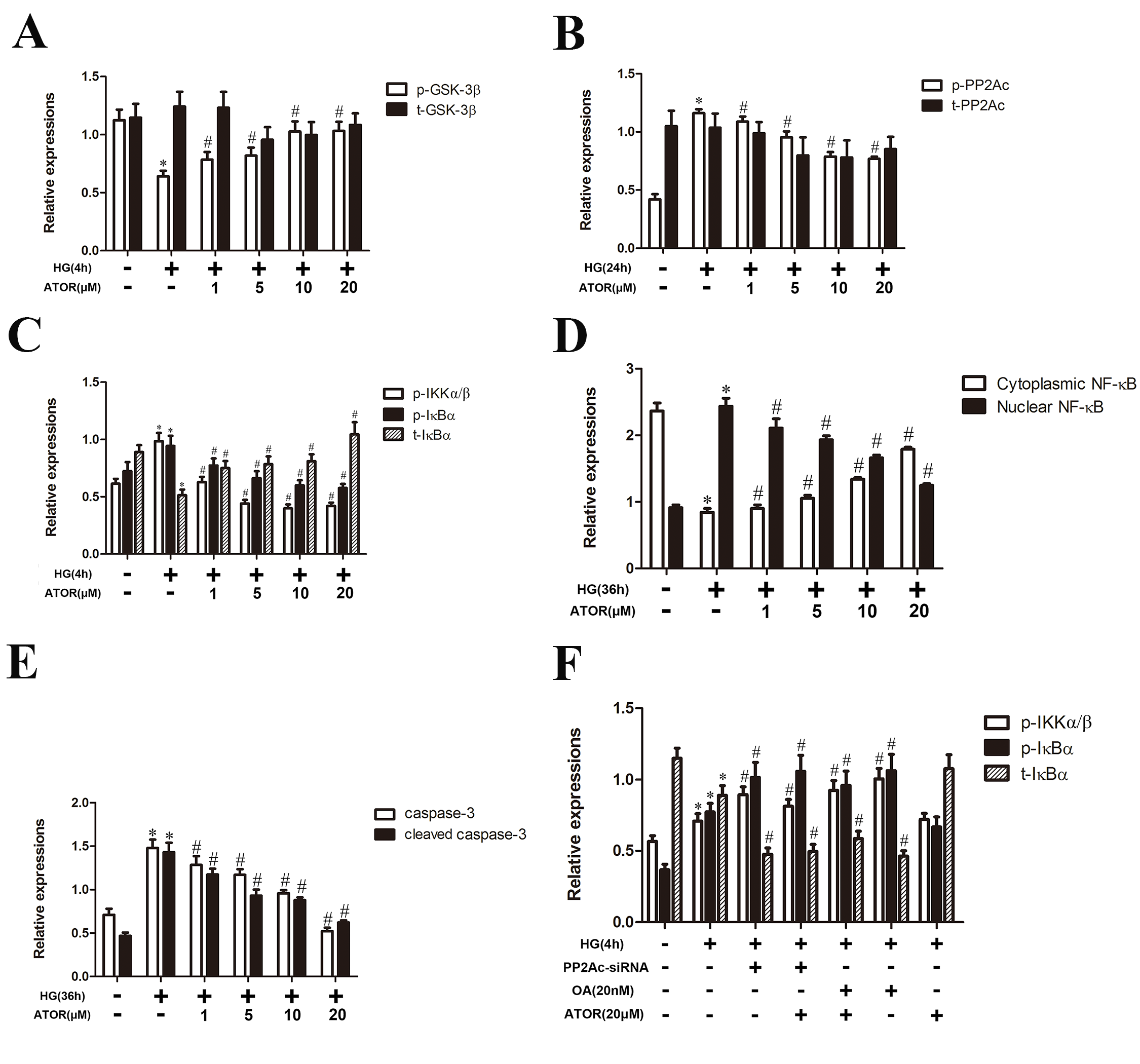

Supplement: S4 Fig — Effects of ATOR on HG-induced activation of the GSK-3β-PP2Ac-IKK-IκBα-NF-κB pathway in H9C2 cells. (A–F) Densitometric quantification of protein bands shown in Fig 5A–5F. All densitometric quantification was performed with Image J. Data are means ± SD and were obtained from at least three independent experiments. *, p<0.05 vs control; #, p<0.05 vs HG. (TIF) [file pone.0166740.s004.tif]

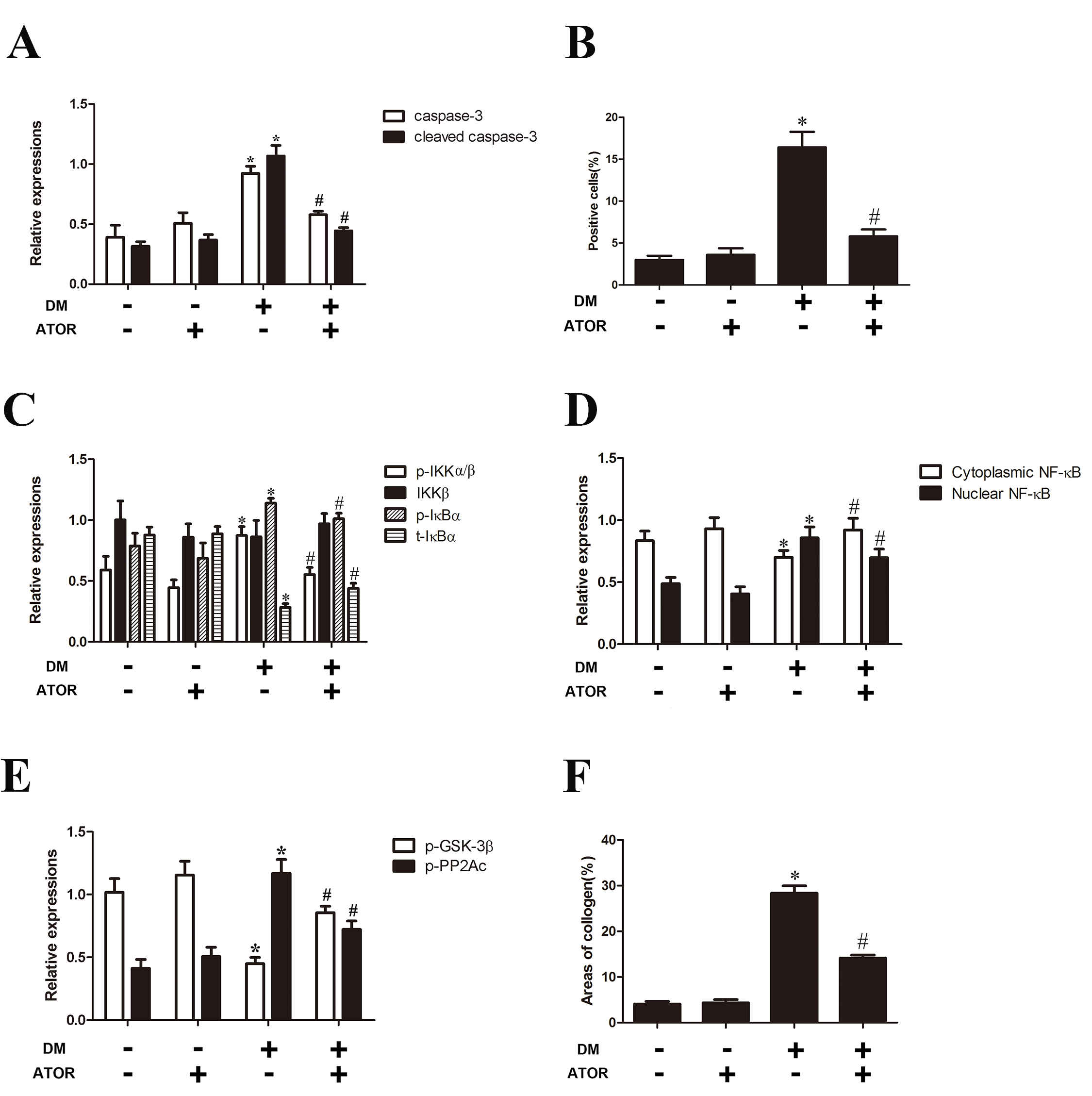

Supplement: S5 Fig — Effects of ATOR on cardiomyocyte apoptosis, the NF-κB pathway and histological alterations in diabetic hearts. (A), (C–E). Densitometric quantification of protein bands shown in Fig 6A and 6C–6E. (B). Quantification of TUNEL-positive cells in diabetic hearts, shown in Fig 6B. (F). Quantification of collagen areas in diabetic hearts, shown in Fig 7B. Data are means ± SD from three independent experiments. *, p<0.05 vs control; #, p<0.05 vs HG. (TIF) [file pone.0166740.s005.tif]
